# Functional and directed connectivity of the cortico-limbic network in mice in vivo

**DOI:** 10.1007/s00429-020-02202-7

**Published:** 2021-01-13

**Authors:** Zeinab Khastkhodaei, Muthuraman Muthuraman, Jenq-Wei Yang, Sergiu Groppa, Heiko J. Luhmann

**Affiliations:** 1 Institute of Physiology, University Medical Center of the Johannes Gutenberg University Mainz, Duesbergweg 6, 55128 Mainz, Germany; 2 Section of Movement Disorders and Neurostimulation, Biomedical Statistics and MULTIMODAL Signal Processing Unit, Department of Neurology, University Medical Center of the Johannes Gutenberg University Mainz, Duesbergweg 6, 55128 Mainz, Germany

**Keywords:** Multi-electrode recordings, Hippocampus, Medial prefrontal cortex, Basolateral amygdala, Nucleus accumbens, Temporal partial directed coherence

## Abstract

Higher cognitive processes and emotional regulation depend on densely interconnected telencephalic and limbic areas. Central structures of this cortico-limbic network are ventral hippocampus (vHC), medial prefrontal cortex (PFC), basolateral amygdala (BLA) and nucleus accumbens (NAC). Human and animal studies have revealed both anatomical and functional alterations in specific connections of this network in several psychiatric disorders. However, it is often not clear whether functional alterations within these densely interconnected brain areas are caused by modifications in the direct pathways, or alternatively through indirect interactions. We performed multi-site extracellular recordings of spontaneous activity in three different brain regions to study the functional connectivity in the BLA–NAC–PFC–vHC network of the lightly anesthetized mouse in vivo*.* We show that BLA, NAC, PFC and vHC are functionally connected in distinct frequency bands and determined the influence of a third brain region on this connectivity. In addition to describing mutual synchronicity, we determined the strength of functional connectivity for each region in the BLA–NAC–PFC–vHC network. We find a region-specificity in the strength of feedforward and feedback connections for each region in its interaction with other areas in the network. Our results provide insights into functional and directed connectivity in the cortico-limbic network of adult wild-type mice, which may be helpful to further elucidate the pathophysiological changes of this network in psychiatric disorders and to develop target-specific therapeutic interventions.

**Supplementary Information:**

The online version contains supplementary material available at 10.1007/s00429-020-02202-7.

## Introduction

Higher cognitive processes and emotional regulation, such as associative learning, episodic memory formation, decision making, threat processing, and anxiety, depend on densely interconnected telencephalic and limbic areas (Simons and Spiers 2003; Sesack and Grace 2010; Preston and Eichenbaum 2013). The ventral hippocampus (vHC), medial prefrontal cortex (PFC), basolateral amygdala (BLA) and nucleus accumbens (NAC) are central structures to compose these cortico-limbic network interactions. Recently, experimental studies in animals and clinical data obtained in humans provided evidence that structural anomalies and aberrant functional coupling in specific connections of this cortico-limbic network may be related to several psychiatric diseases, such as schizophrenia, depression, and post-traumatic stress disorder (Fornito and Bullmore 2015; Hillary and Grafman 2017). It has been recently demonstrated that mice exposed to stress in a model of chronic social defeat display reduced myelin protein content in the NAC and a reduced myelin thickness only in the PFC of stress susceptible mice (Bonnefil et al. 2019). Dysfunction in the vHC to PFC pathway and its reciprocal connectivity to the BLA have been suggested as key mechanisms underlying not only some psychiatric disorders, but also stress regulation and resilience to emotional stress (Godsil et al. 2013; Padilla-Coreano et al. 2019). For example, hippocampus and prefrontal cortex show reduced connectivity in depression (Genzel et al. 2015), abnormal synchrony in schizophrenia (Dickerson et al. 2012) and disruption of plasticity following an acute stress exposure (Rocher et al. 2004). A recent study reported the role of hippocampal-prefrontal theta communication in regulating avoidance behavior (Padilla-Coreano et al. 2019). Furthermore, BLA hyperactivity and NAC hypoactivity have been observed in several mood disorders (for review see Russo et al. 2012). However, it is often not clear whether functional alterations within these densely interconnected brain areas are caused by modifications in the direct pathways, or alternatively through indirect connections. Therefore, the identification of the pathway(s) undergoing pathophysiological changes is essential to develop a specific therapeutic intervention.

Although imaging techniques are powerful methods to monitor the activity from the whole brain (e.g. fMRI) or from hundreds of neurons (e.g. calcium imaging), the temporal resolution of these techniques are inferior as compared to electrophysiological recordings. Furthermore, EEG and MEG have a poor spatial resolution and do not allow recordings from deep brain structures. Thus, intracranial recordings are currently the most powerful method to perform recordings with high spatial and temporal resolution at the network and single cell level even in deep brain structures. For obvious reasons intracranial recordings can be temporally performed in only a rather limited group of patients, cases of Parkinson´s disease during deep brain stimulation (Cagnan et al. 2019) or in pharmaco-resistant epilepsy (Sinha et al. 2017), and in these patients the intracranial electrodes are located in regions relevant to these diseases. Therefore, multi-site recordings simultaneously from many brain regions can be performed only in non-human models. In recent years, the number of in vitro and in vivo animal studies that have performed simultaneous recordings from multiple regions has been increasing. Despite the well-known advantages of in vitro approaches such as cellular and subcellular resolution (e.g. patch-clamp recordings from defined cellular compartments, such as dendrite or presynaptic terminal), experimental control of the extracellular milieu (e.g. to apply drugs in specific concentrations reaching the target structure without restraints by the blood–brain barrier), or well-defined control of presynaptic stimulation (e.g. monosynaptic single-fiber activation), in vitro systems do not fully recapitulate network connectivity (especially of long-range projections), the influence of neuromodulatory systems (e.g. cholinergic or serotonergic modulatory action) and spontaneous network activity (e.g. alpha rhythm in visual cortex). Therefore, we have chosen the in vivo system to analyze functional interactions in the cortico-limbic network consisting of BLA, PFC, NAC and vHC.

Given the anatomical intermingled topographies of functionally distinct neurons in BLA (McGarry and Carter 2017; Beyeler et al. 2018) and NAC (Gangarossa et al. 2013), it is advantageous to record network activity and multi-unit activity at multiple sites. Multi-electrode array (MEA) technology offers the advantage to record the activity (i) from different and even deep brain areas simultaneously, (ii) at multiple sites in the target brain region, and (iii) from the local network (local field potential, LFP) and multi- or single-unit activity (MUA, SUA, respectively). However, depending on the brain regions of interest, such multi-site MEA recordings are difficult to perform in awake or even freely moving animals (but see Hultman et al. 2018).

Here we studied in adult wild-type male *C57BL/6 N* mice the functional and directed connectivity of four brain structures within the cortico-limbic network (vHC–BLA–NAC–PFC) using extracellular MEA recordings. We measured in three areas simultaneously spontaneous LFPs and MUA in a controlled lightly anesthetized state. For each brain region, we determined the dominant frequency and the functional interactions between two connected brain regions. The influence of a third brain region on the interactions between two connected brain areas was determined by quantification of the partial coherence between these three signals. To detect and quantify the functional interactions within and between these networks we used temporal partial directed coherence (tPDC), a novel method previously applied in the analysis of EEG, MEG and fMRI signals (Leistritz et al. 2013; Anwar et al. 2016; Vergotte et al. 2017; Muthuraman et al. 2018). Rather than merely describing mutual synchronicity, as in cross-correlation and coherence analysis, tPDC gives information whether and how two brain structures interact over time and in specific frequency bands via direct feedforward or feedback connections (Baccala and Sameshima 2001; Muthuraman et al. 2018; Pagnotta and Plomp 2018).

Using this approach, we demonstrate that vHC–BLA–NAC–PFC are highly interconnected in their LFP and spike activity. We show the influence of PFC in synchronizing NAC with BLA and vHC, albeit in different extent. We find a region-specificity in the strength of feedforward and feedback connections for each region in its interaction with other areas in the network. To our knowledge this is the first report demonstrating not only the technical feasibility of simultaneous MEA recordings in three brain regions of interest in the lightly anesthetized mouse in vivo, but also the applicability of the directed connectivity (tPDC) method in multi-site brain region recordings and analyses using small animal models.

## Materials and methods

All procedures related to the care and treatments of animals were approved by a local ethics committee (#23 177-07/G 14-1-080) and followed the German and European national regulations (European Communities Council Directive, 86/609/EEC). In total 16 *C57BL6/N* male mice were used, which ranged in age from 2 to 3 months. Nine of these mice were used for simultaneous BLA–NAC–PFC recordings and 7 were used for vHC-NAC-PFC recordings. All recordings have been performed in the right hemisphere.

### Surgical preparation and electrophysiological recordings

Anesthesia was induced by 5% isoflurane and maintained during surgery by a combination of urethane (375 mg/kg, ip), chlorprothixene hydrochloride (2 mg/kg, ip) and isoflurane (1–2%). Lidocain hydrochloride gel 2% was used for local analgesia and atropine (0.3 mg/kg, sc) to reduce bronchial secretions. Animal temperature was kept at 37 °C. A custom-designed head post was mounted to the skull using cyanoacrylate adhesive (Permabond Engineering Adhesives, Eastleigh, UK). As reference we used a wire placed into the cerebellum. During recordings, isoflurane was reduced to 0% to minimize anesthesia-induced unwanted side effects on neural activity and functional connectivity (Williams et al. 2010; Bukhari et al. 2018; Paasonen et al. 2018). Previous fMRI experiments comparing six different anesthesia protocols have demonstrated that the pattern of functional connectivity during urethane anesthesia, even with three times higher urethane concentrations (1250 mg/kg, ip) than used in our experiments, is similar to that observed in awake rodents. Functional connectivity of the default mode network, interhemispheric and complex-network parameters (e.g. correlation coefficients, modularity, clustering coefficients) and thalamocortical connectivity is better preserved in urethane anesthesia as compared to the other anesthetics tested (Paasonen et al. 2018). Depending on the level of anesthesia as assessed by the breathing rate and presence or absence of the pinch toe reflex, additional doses of chlorprothixene hydrochloride (2 mg/kg, ip) were injected (after approximately 3 h). Recordings typically lasted for 5 h.

**Fig. 1:**
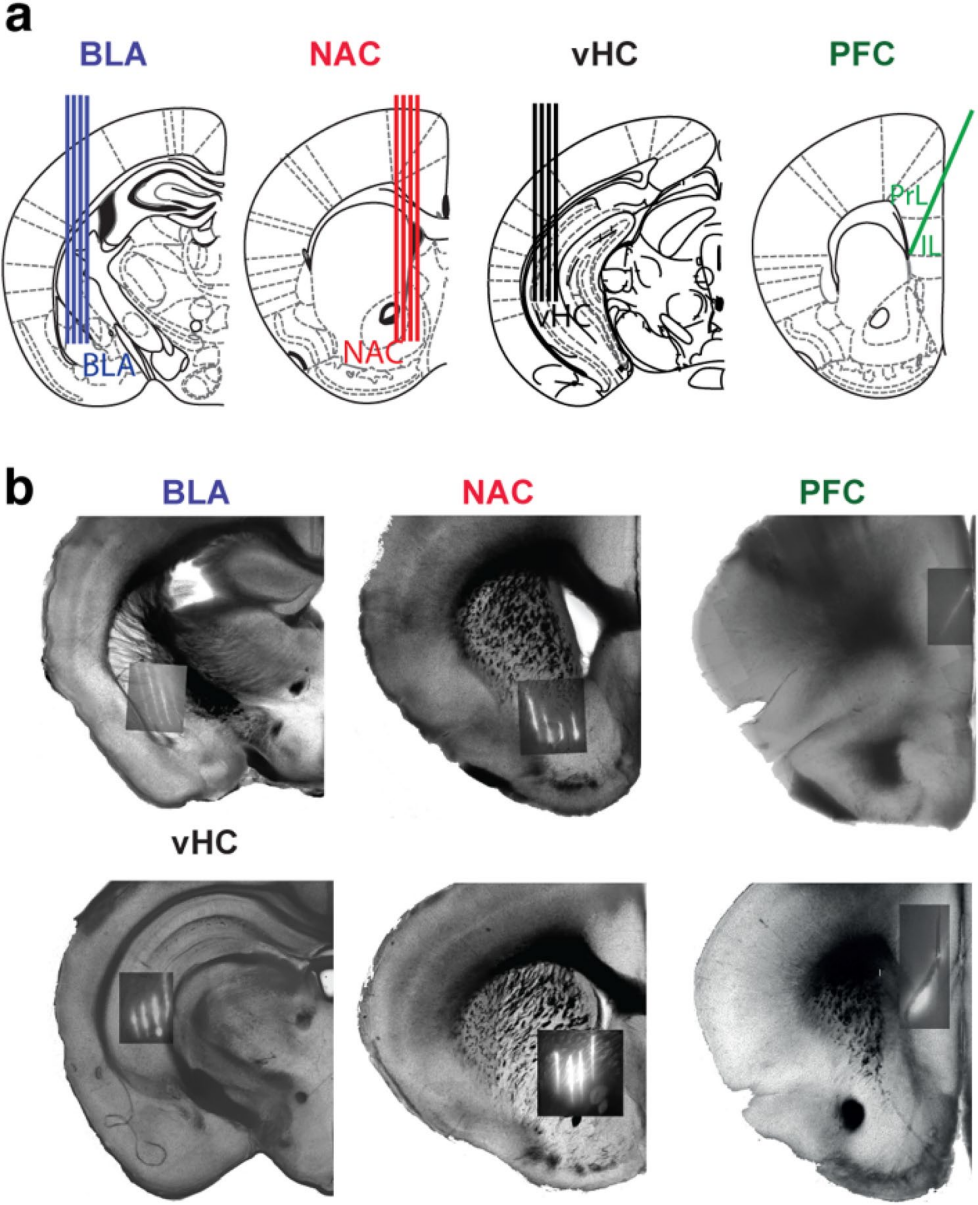
Localization and configuration of MEAs for simultaneous recordings in the cortico-limbic network of the adult mouse in vivo. **a** Schematic illustration of MEAs in BLA, NAC, vHC and PFC. Note that a one-shank MEA for recording in PFC was inserted from the contralateral hemisphere. Sections from Paxinos and Franklin (2001). **b** Photographs of coronal sections showing the track and localization of the MEAs, covered with DiI for the BLA–NAC–PFC configuration (top) and for the vHC–NAC–PFC configuration (bottom). The distance from Bregma: − 1.46, 0.86, 1.98 mm for BLA, NAC and PFC, respectively (top) and − 2.80, 1.10 and 1.70 mm for vHC, NAC and PFC respectively (bottom). In this and following figures, BLA is shown in blue color, NAC in red, vHC in black and PFC in green

We performed extracellular MEA recordings in the vHC–BLA–NAC–PFC network, simultaneously with two technically feasible combinations of MEA localizations in vHC–NAC–PFC or BLA–NAC–PFC (Fig. 1, Suppl. Fig. 1). In each animal we recorded either vHC–NAC–PFC or BLA–NAC–PFC and measured functional connectivity in five distinct pairs: vHC–NAC, vHC–PFC, NAC–PFC, BLA–NAC and BLA–PFC. Using this approach, we were not able to study the vHC–BLA connectivity. Recordings from vHC were obtained through a craniotomy located 3 mm lateral to the midline and 3 mm posterior to Bregma. Recordings from NAC were obtained from 0.8 mm lateral to the midline and 1.1 mm anterior to the Bregma. Due to space limitations, we reached PFC via a contralateral craniotomy located 0.5 mm lateral to the midline and 1.5 mm anterior to the Bregma in a 60° angle. Recordings from BLA were obtained through a craniotomy located 2.8 mm lateral to the midline and 1.5 mm posterior to Bregma. For vHC, BLA and NAC, we used a 32-channel MEA in a 4-shank configuration (A4 × 8-A32, Neuronexus; 200 µm inter-shank spacing), and for PFC we used a one shank 32-channel MEA (A1 × 32-Poly2, Neuronexus). Extracellular signals were recorded at 20 kHz with a ME256-FIA-MPA system or ME2100 system (Multi Channel Systems, Reutlingen, Germany).

### Functional connectivity analyses

To calculate the spike count correlation during spontaneous activity we followed our previous paper (Yang et al. 2013) with modified parameters for surrogate, which includes 100 trials and 100 ms jittering time. The correlation coefficient was considered significant if the values differed from the mean of the surrogate by > 2 SD. To test the neuronal synchrony across multiple areas in the cortico-limbic network as described earlier (Govindan et al. 2006) we measured the LFP coherence between each pair of recording sites. The LFP coherence analysis was computed by band-pass filtering at 0.5–250 Hz. To reduce the adverse effects of volume conduction, we subtracted signals from two neighboring electrodes and obtained bipolar LFPs. Next, for each mouse we selected one representative channel in each area and computed the coherence for each representative channel pair. Finally, we averaged the coherences across all mice. Having *x*(*t*) and *y*(*t*) as the two simultaneously measured signals, we divided the length of each signal (*N*) into multiple segments (*M*) in a length of (*L*), so that *N* = *LM*. To estimate both ordinary and partial coherence it is important to select an optimal length of *L* to maintain both sensitivity and reliability of the estimation. Here, we always used *N* = 6,000,000, *L* = 40,000 and *M* = 150 for coherence analysis. To measure the power spectra *S*_*xx*_ ($\omega$) and *S*_*yy*_ ($\omega$) of the signals *x* and *y,* we performed Fourier transformation of the autocorrelation function in each window. The Fourier transformation of the cross-correlation function of the signal *x* and *y* in each window was considered as cross spectrum of each pair *S*_*xy*_ ($\omega$). Then we averaged the power spectra and the cross spectrum across all the segments for each pair to get the estimate of the same. The coherence was calculated as follows:

$$\hat{C}\left( \omega \right) = \frac{{\left| {\hat{S}_{xy} \left( \omega \right)} \right|^{2} }}{{\hat{S}_{xx} \left( \omega \right)\hat{S}_{yy} \left( \omega \right)}}.$$

The estimate of coherence value is indicated by overcap (Halliday et al. 1995). To test the significance of the coherence at a particular frequency we used confidence limit as an indicator, which was calculated at the 100% $\alpha$ as given by $1 - \left( {1 - \alpha } \right)^{{1/\left( {M - 1} \right)}}$(Halliday et al. 1995; Govindan et al. 2006) where $\alpha$ is set to 0.90, so the confidence limit is $1 - 0.1^{{1/\left( {M - 1} \right)}}$. Two-time series were considered as “correlated” if the coherence value was above this confidence limit and “uncorrelated” if the coherence value was below this confidence limit.

Similarly, we measured the partial coherence between the three signals *x*(*t*), *y*(*t*) and *z*(*t*) as follows:

$$\widehat{{C_{xy|z} \left( \omega \right)}} = \frac{{\left| {\widehat{CY}_{xy} \left( \omega \right) - \widehat{CY}_{xz} \left( \omega \right)\widehat{CY}_{zy} \left( \omega \right)} \right|^{2} }}{{\left( {1 - \hat{C}_{xz} \left( \omega \right)} \right)\left( {1 - \hat{C}_{zy} \left( \omega \right)} \right)}},$$

where the magnitude of *CY*_*xy*_ ($\omega$) is called coherency between the two signals *x* and *y*. Again, the overcap indicates the estimate of that value (Halliday et al. 1995).

### Time-resolved partial directed coherence (tPDC)

Using time–frequency causality we cannot only focus on a particular frequency itself but can also analyze the time-dynamics of the causality at that frequency. Based on state-space modelling, the time–frequency causality estimation method of tPDC relies on dual-extended Kalman filtering (DEKF) (Wan and Nelson 2001). These results are an estimate of the time-varying dependent autoregressive (AR) coefficients: one EKF estimates the states and feeds this information to the second DEKF, which estimates the model parameters and back propagates this information to the initial EKF. By concurrently using two Kalman filters working in parallel with one another, it is possible to estimate both states and model parameters of the system at each time instant. After estimating the time-varying multivariate (MVAR) coefficients, the next step is to use those coefficients for the calculation of causality between the time series. Since DEKF yields the time varying MVAR coefficients at each time point, we can calculate the partial directed coherence (PDC) at each time point as well. Afterwards, a time–frequency plot using all PDCs can be concatenated to produce a time–frequency plot. The precise distribution of the MVAR coefficients is not known; we used the surrogate method called bootstrapping (Kaminski et al. 2001) to check for the significance of the results. This method is based on the random shuffling of the subjected time series and hence it is data driven. In short, it divides the original time series into smaller non-overlapping windows and randomly shuffles the order of these windows to create a new time series. The MVAR model was fitted to this shuffled time series. This process was repeated 1000 times and their average are calculated. The resulting 95th percentile value of the null distribution is the significance threshold value for all connections. Additionally, we applied time reversal technique (Haufe et al. 2013) as a second significance test on the connections already identified by tPDC using data driven bootstrapping surrogate significance test.

### Histology

For post hoc identification of the recording sites, we coated each electrode with fluorescent lipophilic dye (DiI, D282, Invitrogen). After recordings, mice were transcardially perfused under xylazine hydrochloride anesthesia (30 mg/kg) with 0.1 M phosphate buffer (PB), followed by 4% paraformaldehyde (PFA). Brains were removed and post-fixed in 4% PFA at 4 °C overnight and after rinsing 3 times with 0.1 M PB transferred to 30% sucrose in 0.1 M PB solution. Using a freezing microtome (Leica, Wetzlar, Germany) brains were sliced at 200 µm thickness and were inspected for the presence of the tracers using a fluorescent microscope (Fig. 1b).

### Statistics

Statistical analyses were performed in MATLAB and GraphPad Prism. To investigate the direction- and region-specificity of the connections we performed an ANOVA with the within-subject factor direction of connectivity (*x*–*y* vs. *y*–*x*; *x*–*z* vs. *z*–*x*; *y*–*z* vs. *z*–*y*), and the between-subject factors region (*xy* vs. *xz*; *xy* vs. *yz*; *xz* vs. *yz*) using MATLAB. All ANOVA analysis were followed by post hoc using *multcompare* function in MATLAB. For the ANOVA results that showed interaction between factors, a paired *t* test was performed following ANOVA. To test the difference in cross-correlations we performed a Mann–Whitney *U* test in GraphPad Prism.

## Results

### Spontaneous activity in cortico-limbic network

**Fig. 2:**
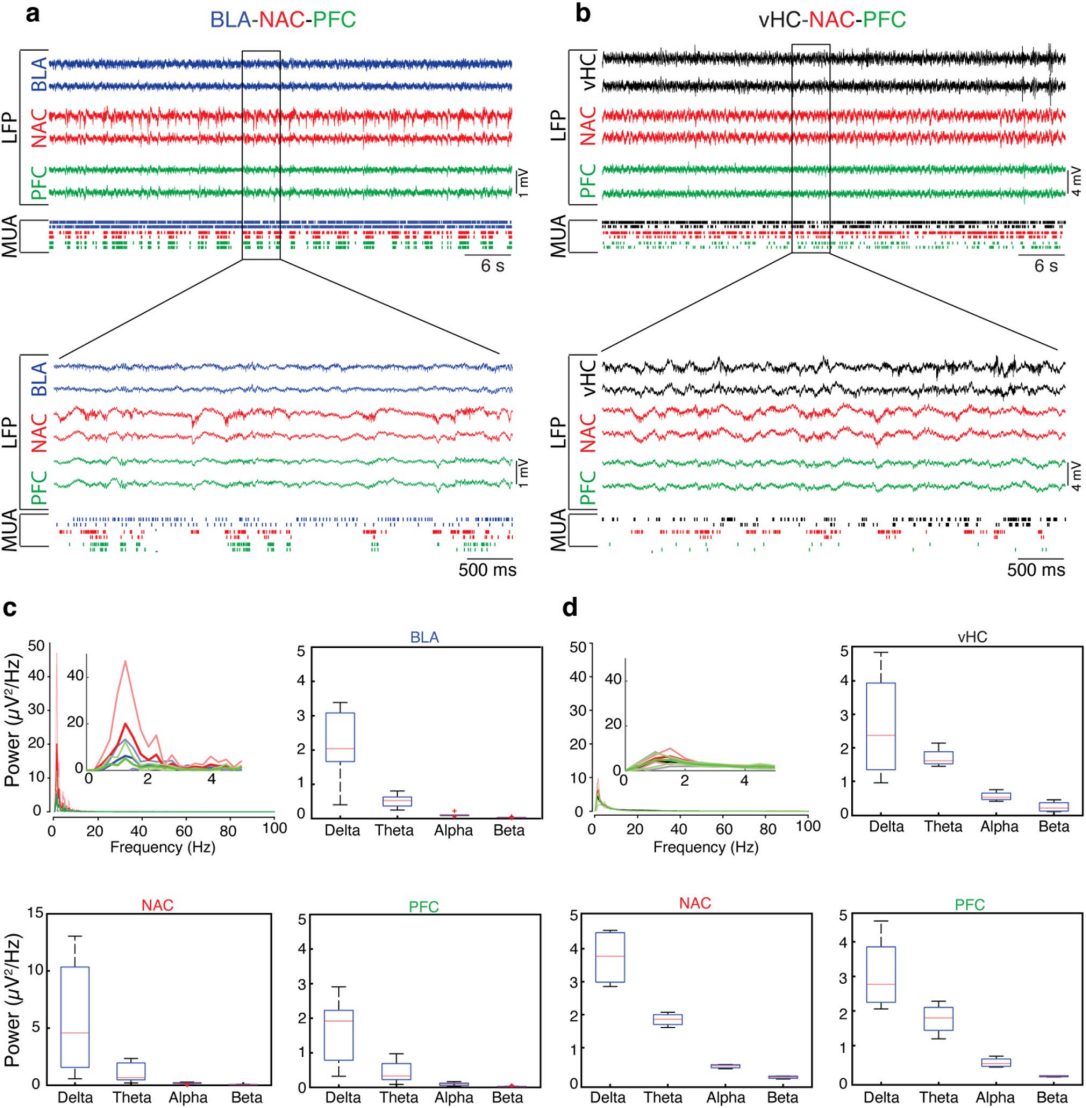
Simultaneous recordings of spontaneous activity in three brain regions. **a** Local field potentials (LFPs) and multi-unit activity (MUA) recorded simultaneously in BLA, NAC and PFC. Lower traces show recordings at higher temporal resolution. **b** Same as in (**a**) for simultaneous recordings in vHC, NAC and PFC. **c** Power spectra of spontaneous LFP activity in BLA, NAC and PFC averaged across all mice (*n* = 9). Thick lines represent mean and thin lines represent ± SD. In boxplots, frequency bands are definded as delta from 0 to 3 Hz, theta from 4 to 7 Hz, alpha from 8 to 15 Hz, and beta from 16 to 31 Hz. **d** Same as in (**c**), but for simultaneous recordings in vHC, NAC and PFC in 7 mice. Please note the different scale of NAC boxplot in (**c**) as compared to other boxplots. In this and following figures, thin red lines in boxplots indicate the medians and red crosses show outliers

Spontaneous activity in the cortico-limbic network was characterized by stable neural activity in all recorded regions over a long time period such that the average firing rates at the beginning and at the end of the experiments were comparable (6.36 ± 3.32 vs. 7.63 ± 5.97 Hz in BLA, 21.78 ± 19.06 vs. 26.23 ± 18.61 in NAC, 4.85 ± 3.27 vs. 5.21 ± 3.92 in PFC, 7.75 ± 7.9 vs. 8.03 ± 8.19 in vHC, *n* = 992 channels from 16 animals, mean ± SD; Fig. 2a, b). Given the previous data on cortico-limbic network synchrony in lower frequencies (Hultman et al. 2016), we concentrated our further analysis on frequencies below gamma (all frequencies less than 32 Hz). Defining the frequency bands as delta = 0.5–3 Hz, theta = 4–7 Hz, alpha = 8–15 Hz, beta = 16–31 Hz and gamma = 32–100 Hz, LFPs in all four brain regions showed a high power in lower frequencies. The strongest power of LFP in BLA, NAC and PFC were observed in the delta band and in vHC in both delta and theta bands. In the BLA–NAC–PFC configuration (Fig. 2c) the median power in the delta band was 2.04 (BLA), 4.58 (NAC), 1.92 µV^2^/Hz (PFC), and in theta band 0.52 (BLA), 0.66 (NAC), 0.33 µV^2^/Hz (PFC). Higher frequencies showed lower power with alpha 0.09 (BLA), 0.19 (NAC), 0.09 µV^2^/Hz (PFC), and beta 0.02 (BLA), 0.02 (NAC), 0.02 µV^2^/Hz (PFC). In the vHC-NAC-PFC configuration (Fig. 2d) the median power of delta was 2.37 (vHC), 3.76 (NAC), and 2.77 µV^2^/Hz (PFC), and theta 1.61 (vHC), 1.86 (NAC), 1.8 µV^2^/Hz (PFC). The median power was lower for alpha 0.52 (vHC), 0.45 (NAC), 0.48 µV^2^/Hz (PFC), and for beta 0.2 (vHC), 0.11 (NAC), 0.12 µV^2^/Hz (PFC). These findings are not only in agreement with previous reports (Dzirasa et al. 2011a; Hultman et al. 2016; Padilla-Coreano et al. 2019), but also indicate the stability of all recording sites over experimental sessions lasting up to 5 h, as the average firing rates are relatively constant during the entire recording session.

### Functional connectivity of MUA within the cortico-limbic network

**Fig. 3:**
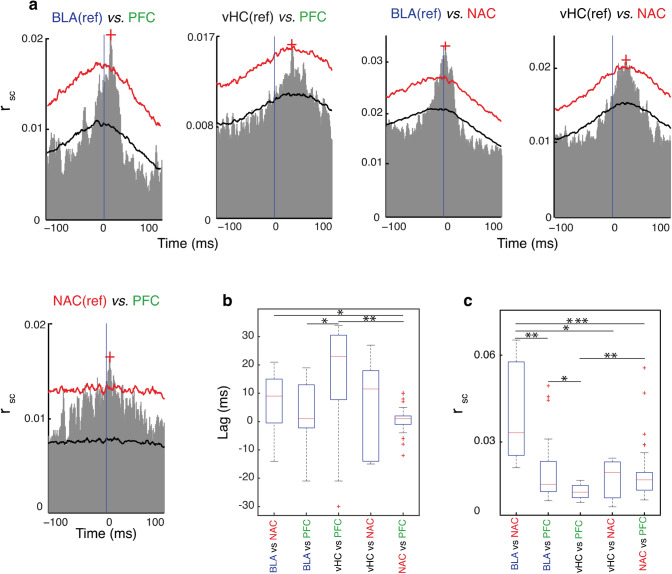
Spike count cross-correlations within the cortico-limbic network. **a** Representative cross-correlations between two brain regions. In all graphs black curves correspond to mean of surrogate, red curves correspond to mean standard deviation of surrogate and red crosses correspond to the maximum strength of correlation and “ref” stands for reference channel. **b** Comparison of the lag time of each region with two other connected regions averaged across all mice (*n* = 9 for BLA-NAC-PFC, *n* = 7 for vHC-NAC-PFC recording condition). In the boxplot * represents *p* < 0.05 and **p < 0.002. **c** Comparison of the maximum correlation strength of each region with two other connected regions averaged across all mice (*n* = 9 for BLA-NAC-PFC, n = 7 for vHC-NAC-PFC recording condition). In the boxplot * represents *p* < 0.05, **p < 0.001, ***p < 10^–4^

To measure the functional connectivity within the cortico-limbic network, we computed cross-correlation analyses (spike count correlation: *r*_SC_) of MUA during spontaneous activity for the five paired brain regions (Fig. 3). Figure 3a shows examples of cross-correlations for five paired regions. The cross-correlation functions of BLA–PFC interactions showed the largest peak at a positive lag of 10–20 ms, which means BLA (reference channel) is driving PFC (target channel) (Fig. 3a, top left panel, median across 9 mice 1 ms, Fig. 3b). We also found a correlation between the number of spikes in BLA and NAC, which occurred at a lag of 0–10 ms, indicating BLA is leading NAC (Fig. 3a, median across 9 mice 9 ms, Fig. 3b). NAC and PFC showed a clear peak of correlation occurring at a positive lag of 0–10 ms, demonstrating that NAC drives PFC (Fig. 3a, lower panel, median across 9 mice 1 ms, Fig. 3b). In vHC–NAC–PFC recordings, the cross-correlation analysis revealed that vHC is correlated with PFC and NAC. The vHC–NAC and vHC–PFC cross-correlations illustrate that vHC drives both NAC and PFC (Fig. 3a, top, median across 7 mice 11.5 for vHC–NAC and 23 ms for vHC–PFC, Fig. 3b). It should also be noted that for each pair of the five tested connections, the time lag of cross-correlation peak was not always consistent across different channel combinations, such that in a given paired region we observed negative, positive and/or zero time lags in different channel combinations (Fig. 3b). Comparing the lag time of correlation between different pairs, we found that the BLA–NAC time lag of correlation is significantly longer than NAC-PFC (*p* = 0.04, Mann–Whitney *U* test; Fig. 3b). In addition, the lag time of correlation between vHC and PFC is significantly longer than BLA–PFC correlation’s lag time (*p* = 0.01, Fig. 3b) and also NAC–PFC correlation’s lag time (*p* < 0.002, Fig. 3b). Although the medians of lag time of cross-correlation in all five pairs are in positive range the variability of the results was rather high as evident in large error bars. Under this condition, resolving the directionality based on merely cross-correlation analysis is difficult (Bastos et al. 2018) and therefore, we used another method to determine the direction of functional connectivity (see below). In summary, a substantial number of channels in all five pairs showed significant correlations during spontaneous activity.

Next, we asked whether the correlation strength of a given region with the second area is different than that of the third region. To address this question, we compared the *r*_SC_ values of each pair (e.g. *r*_SC_ of BLA–PFC) with that of its corresponding third region (i.e. *r*_SC_ of BLA–NAC) and averaged across all mice. We found that for BLA, the correlation in the BLA–NAC pair is significantly stronger than the correlation in the BLA–PFC pair (*p* < 0.001, Mann–Whitney *U* test; Fig. 3c). For NAC, we found that the BLA–NAC correlation is significantly stronger than the NAC–PFC correlation (*p* < 10^–4^, Mann–Whitney *U* test; Fig. 3c). In vHC–NAC–PFC recordings, we found that the strength of correlation in NAC–PFC is stronger than the vHC-PFC correlation (*p* < 0.001, Mann–Whitney *U* test; Fig. 3c). Comparing the correlation between BLA and vHC with the other areas, we noted that the correlation in BLA–PFC is stronger than the vHC–PFC correlation (*p* = 0.02, Mann–Whitney *U* test; Fig. 3c) and the correlation in BLA–NAC is stronger than the vHC–NAC correlation (*p* = 0.01, Mann–Whitney *U* test; Fig. 3c). Altogether, the spike count cross-correlation results demonstrate that within the cortico-limbic network the BLA–NAC correlation is the strongest among all four connections studied. Furthermore, BLA makes a stronger connection with NAC and PFC than vHC does with these two regions.

### Ordinary and partial coherence of LFPs during spontaneous activity

**Fig. 4:**
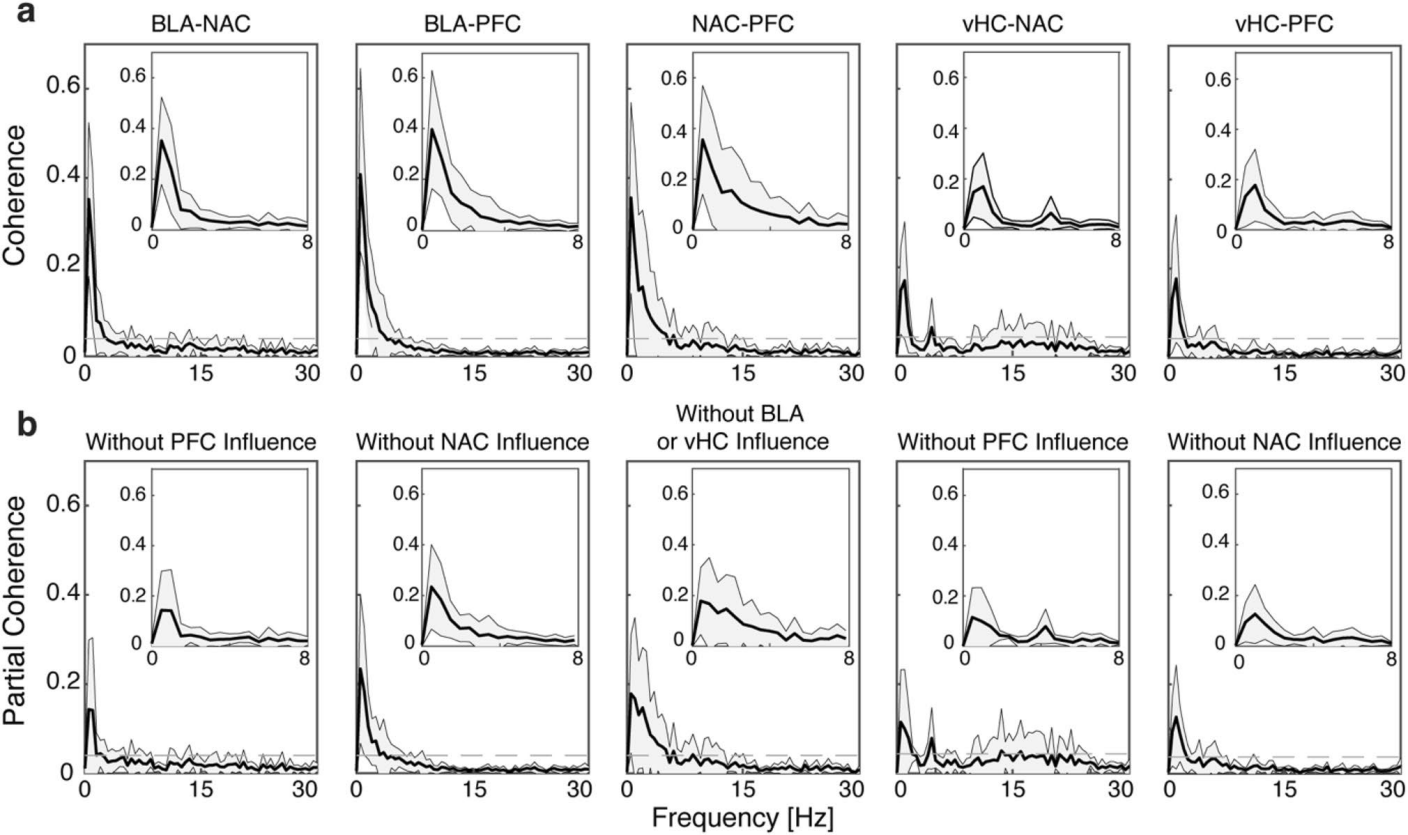
Ordinary coherence and partial coherence in the BLA-NAC-PFC and the vHC–NAC–PFC network. **a** Ordinary coherence plots for five tested pairs. Average of ordinary coherence in BLA-NAC (*n* = 9), BLA–PFC (*n* = 9), NAC–PFC (*n* = 9), vHC-NAC (*n* = 7) and vHC–PFC (*n* = 7). **b** Same as in a, but for partial coherence after removing the influence of another brain region. Note changes in peak coherence as compared to plots in (**a**). In all ordinary and partial coherence plots solid lines show the means and shaded areas show the standard deviations of coherence averaged across all mice. Dashed lines show the confidence limit of significance for coherence

Next, we asked whether and how areas of the cortico-limbic network are also correlated in their LFP activity (Fig. 4). To address this question, we measured the coherency for BLA–NAC, BLA–PFC, NAC–PFC (*n* = 9 animals), and vHC–NAC and vHC–PFC (*n* = 7) for each mouse and then averaged it across all animals (Fig. 4a). We found that BLA and NAC are significantly coherent at ~ 0.5 Hz (0.35 ± 0.17, mean ± SD; Fig. 4a, left). Similarly, BLA and PFC showed a significant coherence with similar pattern as that observed in BLA–NAC. The maximum coherence of BLA–PFC occurred at 0.5 Hz (0.4 ± 0.23, Fig. 4a, second panel from left). We also found a significant coherency in NAC–PFC, which tended to be strongest at lower frequencies with a peak occurring at 0.5 Hz (0.35 ± 0.21; Fig. 4a, middle). In vHC–NAC–PFC recordings, vHC and NAC showed the maximum coherency at 1 Hz (0.16 ± 0.13, Fig. 4a, second plot from right). As expected, vHC and PFC also were significantly coherent and showed a maximum peak occurring at 1 Hz (0.17 ± 0.13, Fig. 4a, right). These results demonstrate coherence between all five pairs, although the coherence strength is different across pairs such that BLA–PFC coherence is the strongest and vHC–NAC coherence is the weakest.

Next, we tested whether the observed coherence in each pair is due to common influence of a third area. We measured the partial coherence for each pair after removing the influence of the third area and averaged it across all mice (Fig. 4b). Left panel of Fig. 4b shows partial coherence between BLA and NAC when the contribution of PFC as a potential common influence has been removed (BLA–NAC/PFC). When comparing ordinary coherence and partial coherence for the BLA–NAC pair, we found that the peak of coherence between BLA and NAC decreased by 60% from 0.35 ± 0.17 to 0.14 ± 0.15 after removing the contribution of PFC (Fig. 4b, left). This result demonstrates the powerful impact of the PFC on the BLA-NAC coherency, although it cannot fully account for the BLA–NAC connection, as their partial coherence still remains significant even after removing the PFC common influence. Similarly, we computed partial coherence for the BLA–PFC pair by excluding the NAC influence (Fig. 4b, second panel from left). We found that the maximum coherence of BLA-PFC decreased by 42.5% from 0.4 ± 0.23 to 0.23 ± 0.16 after removing the NAC influence. Since BLA-PFC coherence after removing NAC contribution still remains significant, it cannot fully be explained by the NAC influence. This means that despite the remarkable contribution of NAC in BLA-PFC coherence, the BLA–PFC coherence is mainly a result of either direct communication between BLA and PFC or indirect via other brain regions, which were not recorded in our experiments. Finally, we removed the BLA to measure its influence on the coherency between NAC and PFC (Fig. 4b, middle panel). We found that the peak of partial coherence in the NAC–PFC pair decreased by approximately 50% from 0.35 ± 0.21 to 0.17 ± 0.13. This finding demonstrates the important implication of BLA in synchronizing the NAC and PFC areas. However, the NAC–PFC coherency cannot fully be explained by BLA influence, as it remained significant even after removing the BLA influence. Similar to BLA–NAC–PFC recordings, we computed partial coherence for the vHC–NAC and vHC–PFC pairs by excluding the PFC and NAC influence, respectively (Fig. 4b, right panels). We found that the maximum coherence of vHC–NAC decreased by approximately 31% from 0.16 ± 0.13 to 0.11 ± 0.11 after removing the PFC influence (Fig. 4b, second plot from right). Despite of this reduction the vHC–NAC coherence remained significant and therefore, PFC influence can only partly describe the vHC–NAC coherence. When comparing ordinary coherence and partial coherence for vHC–PFC pair, we noticed that the peak of coherence between vHC and PFC declined by 30% from 0.17 ± 0.13 to 0.12 ± 0.11 after removing the NAC influence. This reduction demonstrates the NAC contribution in synchronizing vHC and PFC, however, although the majority of vHC and PFC coherency is preserved without NAC involvement. Our results in partial coherence analysis suggest that within the cortico-limbic network an individual region plays an important role in synchronizing other regions although the contribution on this synchronization is different and varies from 30% (influence of NAC on vHC–PFC synchrony) to 60% (influence of PFC on BLA–NAC synchrony).

### Time resolved partial directed coherence during spontaneous activity

**Fig. 5:**
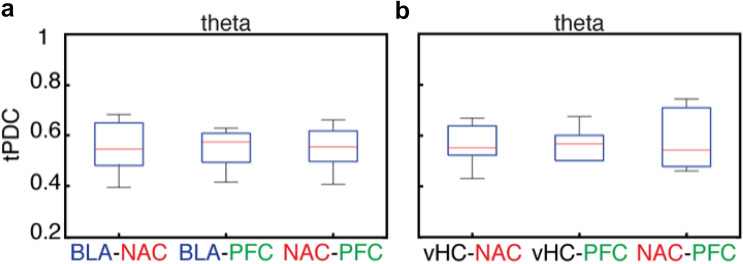
Comparison of areas’ interactions in the BLA-NAC-PFC (**a**) and vHC-NAC-PFC (**b**) recording configuration for theta activity only. **a** Comparison between the total tPDC values for BLA-NAC (left), BLA-PFC (middle) and NAC-PFC (right) in theta band averaged across all mice (*n* = 9). **b** Comparison between the total tPDC values for vHC-NAC (left), vHC-PFC (middle) and NAC-PFC (right) in theta band averaged across 7 mice

**Fig. 6:**
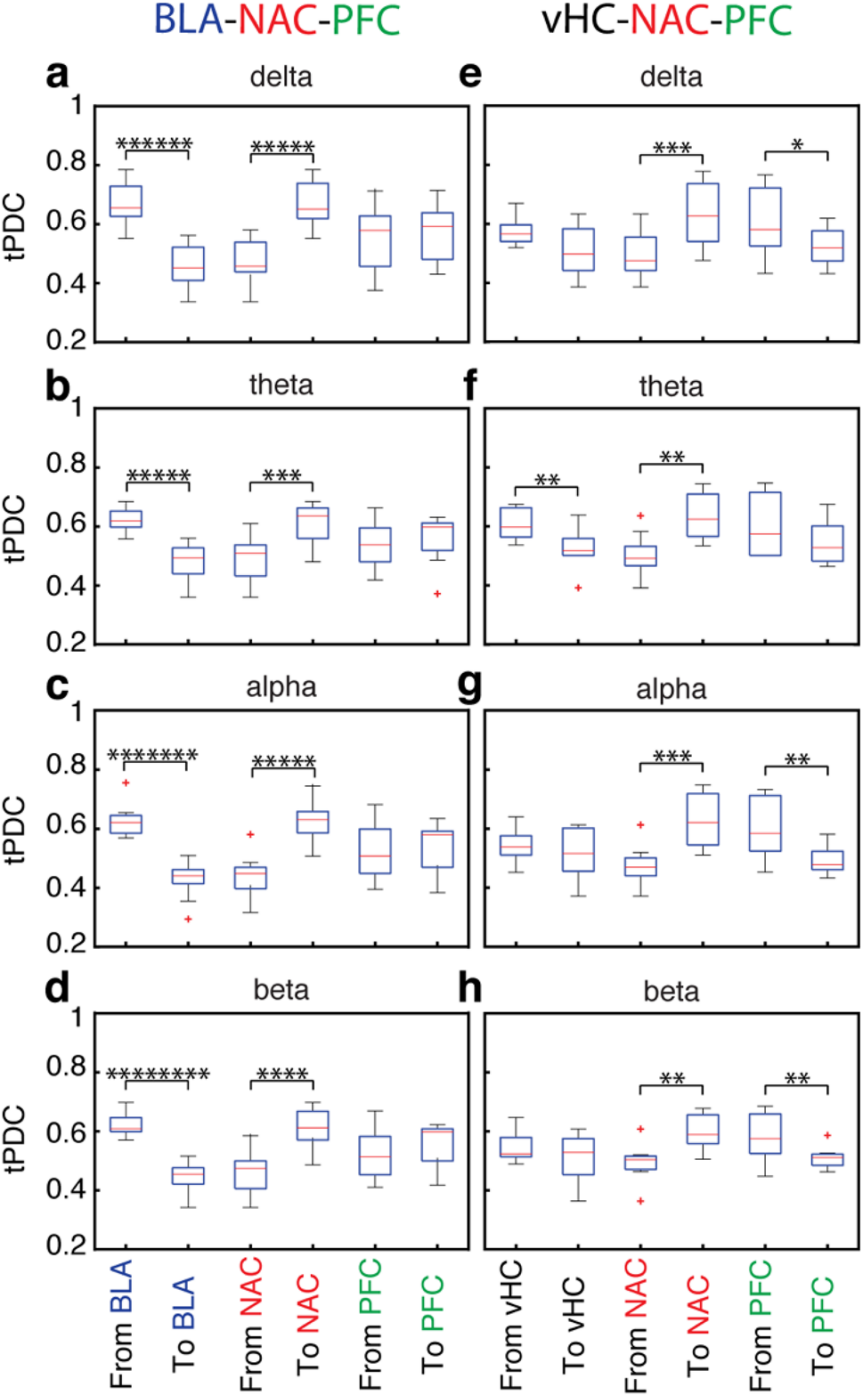
Comparison of inward and outward information flow in BLA, vHC, NAC and PFC in all frequency bands in the BLA-NAC-PFC (left, *n* = 9 mice) and vHC-NAC-PFC recording configuration (right, *n* = 7). **a**–**d** Information flow for BLA (left) and NAC (middle) in delta (**a**), theta (**b**), alpha (**c**) and beta band (**d**). Information flow for PFC in (right, all p > 0.05). **e**–**h**, inward and outward information flow for vHC in all frequency bands in the vHC-NAC-PFC recording configuration (left). Information flow for NAC (middle) and for PFC (right). In all panels * represents *p* < 0.05, **p < 0.001, ***p < 10^–4^, ****p < 10^–5^, ***** *p* < 10^–6^, ******p < 10^–7^, *******p < 10^–8^ and ********p < 10^–9^

**Fig. 7:**
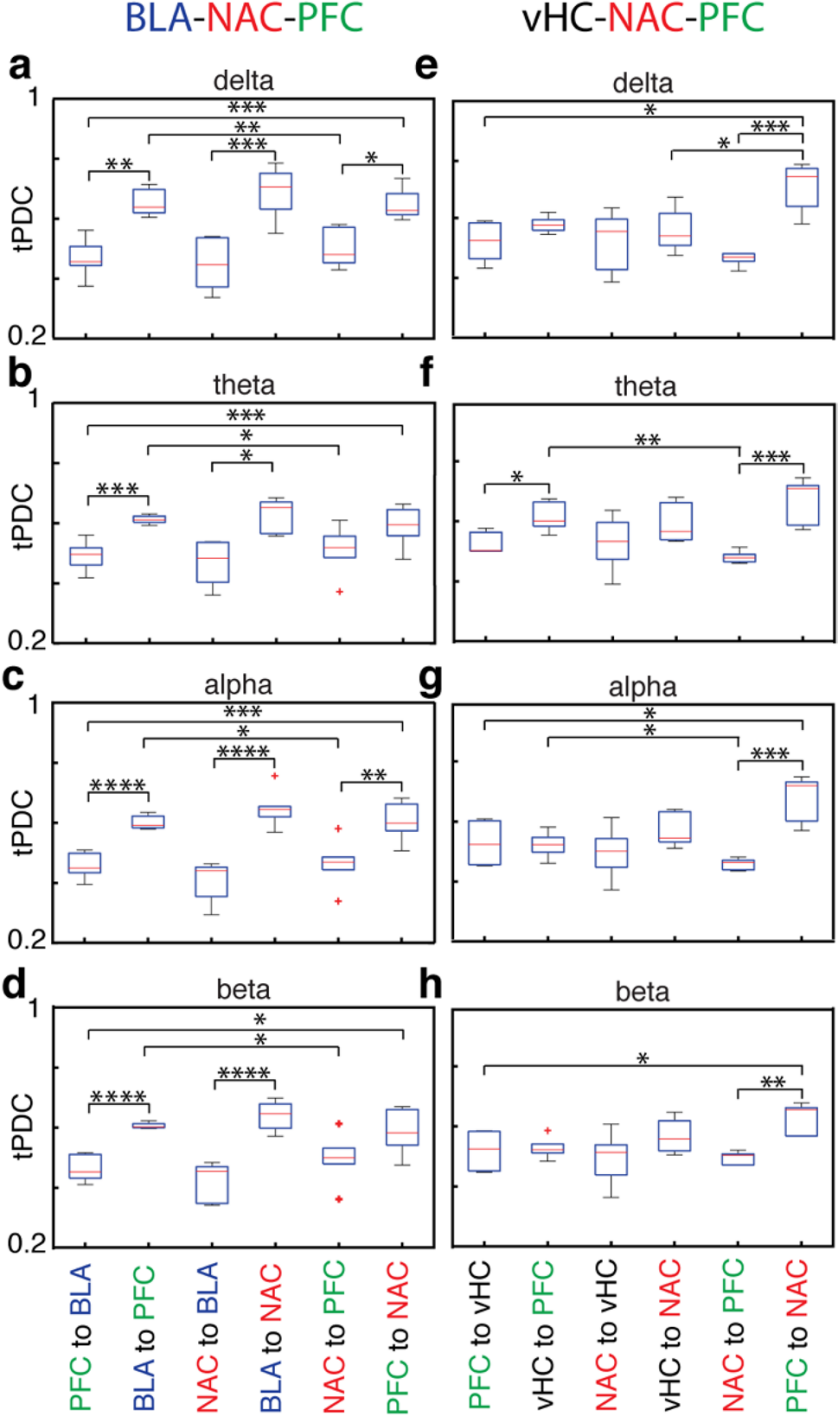
Region-specific directionality of information flow in different frequency bands in the BLA-NAC-PFC (left) and vHC-NAC-PFC network (right). For further details, see Fig. 6 and text

We used tPDC to quantify the directed connectivity in five different frequency bands for both the BLA-NAC-PFC and the vHC-NAC-PFC network (Figs. 5, 6 and 7). Since the LFP power in the gamma range was rather small, we focused on delta to beta frequencies. In the BLA-NAC-PFC network, every single area interacted with the two others without any significant region-preferences. This is illustrated in Fig. 5a exemplary for the activity in the theta band. However, when we took the direction of information flow into account, we observed significant direction preferences in information flow for all three regions in multiple frequencies. We found that in all frequencies bands the tPDC values for BLA outputs were significantly higher than the tPDC values for BLA inputs (ANOVA, main effect, *p* < 10^–7^ [delta], *p* < 10^–6^ [theta], *p* < 10^–8^ [alpha] and *p* < 10^–9^ [beta], Fig. 6a–d). This directionality of the BLA in interaction with others, however, was not region specific, as the tPDC values for BLA to PFC were comparable to tPDC values for BLA to NAC and tPDC values for NAC to BLA were similar to tPDC values for PFC to BLA (*p* > 0.05, Fig. 7a–d). The directionality pattern of NAC interactions with others was opposite to what we observed in the BLA, as the strength of information flow from NAC to BLA and PFC was significantly weaker than the strength of information flow from BLA and PFC to NAC (ANOVA, main effect, *p* < 10^–6^ [delta], *p* < 10^–4^ [theta], *p* < 10^–6^ [alpha] and *p* < 10^–5^ [beta], Fig. 6a–d). Similar to the BLA, we did not find a significant region specificity in directionality neither in inputs to NAC from BLA and PFC, nor in the outputs from NAC to BLA and PFC (*p* > 0.05, Fig. 7a-d). In the PFC, the total PFC output was comparable with the total input to the PFC (*p* > 0.05, Fig. 6a–d). However, the PFC showed a region-specificity in the direction of information flow for interacting with NAC and BLA (Fig. 7a–d). Indeed, the tPDC values for PFC to BLA were significantly lower than tPDC values for BLA to PFC (ANOVA, direct extension of main effect, *p* < 10^–3^ [delta], *p* < 10^–4^ [theta], *p* < 10^–5^ [alpha] and *p* < 10^–5^ [beta], Fig. 7a–d). In contrast, the tPDC values for PFC to NAC were significantly higher than the tPDC values for NAC to PFC (ANOVA, direct extension of main effect, *p* = 0.01 [delta] and *p* < 10^–3^[alpha], Fig. 7a, c). These results not only demonstrate the direct connectivity between BLA, NAC and PFC in distinctive frequency bands, but also suggest a target-specificity in the strength of information flow between these regions. Indeed, in the BLA–NAC–PFC network, BLA strongly transmits information to the other regions, while NAC predominantly acts as a target region and PFC regulates BLA–NAC–PFC network.

In the vHC–NAC–PFC network every single area interacted with two others without any significant region-preferences, as shown exemplary in Fig. 5b for the theta frequency. We found that the tPDC values for vHC outputs in theta band were significantly higher than the tPDC values for vHC inputs (ANOVA, main effect, *p* < 10^–3^; Fig. 6f). However, there was no significant difference for vHC interactions with NAC or with PFC in other frequency bands (Fig. 7e–h). In all frequencies we found a significantly higher information flow in the inputs to NAC as compared to the outputs from NAC (ANOVA, main effect, *p* < 10^–4^ [delta], *p* < 10^–3^ [theta], *p* < 10^–4^ [alpha], *p* < 10^–3^ [beta], Fig. 6e–h). This directionality in NAC interaction with two other regions was region-specific only in delta band where the tPDC values for PFC to NAC were significantly higher than the tPDC values for vHC to NAC (ANOVA, direct extension of main effect, *p* = 0.02; Fig. 7e). Similar to vHC and NAC, we found a directionality in information flow in interaction of PFC with vHC and NAC, such that in all frequency bands except for theta the tPDC values for total PFC inputs was significantly lower than total PFC output (ANOVA, main effect, *p* = 0.01 [delta], p < 10^–3^ [alpha], p < 10^–3^ [beta], Fig. 6e, g, h). In the theta band the tPDC values for vHC to PFC were significantly higher than the tPDC values for PFC to vHC (ANOVA, direct extension of main effect, *p* = 0.01, Fig. 7f). In contrast, in the other frequency bands, there was no significant difference between the strength of information flow for vHC to PFC and for PFC to vHC (Fig. 7e, g, h). Finally, in all frequencies the tPDC values for PFC to NAC were significantly higher than NAC to PFC (ANOVA, direct extension of main effect, *p* < 10^–4^ [delta], *p* < 10^–4^ [theta], *p* < 10^–4^ [alpha], *p* < 10^–3^ [beta], Fig. 7e–h). In summary, these results indicate direct connectivity between vHC, NAC and PFC with a clear frequency-specific role for vHC. In the vHC–NAC–PFC network, vHC act as network driver only in theta band, whereas in the other frequency bands more an interplay between these nodes was found.

## Discussion

Using MEAs, we recorded in lightly anesthetized adult mice the spontaneous activity simultaneously at multiple sites of the cortico-limbic network and analyzed the direction and the strength of the functional interactions by spike count cross-correlations and by time-resolved partial directed coherence (tPDC) calculations. Given our light anesthesia protocol for which we needed to use only one-third of the common dosage of urethane without repetition, we assume that the adverse effects of anesthesia on our results are rather small. This assumption is supported by the similarity of our results with previous observations obtained in awake rodents (see below). Our results demonstrate that BLA, NAC, PFC and vHC, as the key structures of the cortico-limbic network, are highly interconnected and synchronized during their spontaneous activity. Determining the influence of each region in network synchrony and measuring the strength of feedforward and feedback connections, we identified the PFC as a powerful regulator for network synchrony, the BLA as a major source of information flow, NAC as a hub for converging information flow from other network structures and vHC as a main controller for PFC in theta band. These findings may contribute to a better understanding of the physiological and pathophysiological interactions in the cortico-limbic network.

### Region-specific spontaneous activity

All four brain regions revealed the strongest spontaneous activity in the delta and theta frequency range and in the Fourier spectrum a gradual power decrease from alpha over beta to gamma (Fig. 2). Although the presence of delta activity is a strong indicator for the effects of anesthesia, delta and theta activity are also prominent rhythms of the hippocampus and prefrontal cortex of awake rats during a working-memory task (Fujisawa and Buzsáki 2011). Hippocampal respiration rhythms between 2 and 12 Hz and driven by the olfactory bulb have been demonstrated in awake mice (Nguyen et al. 2016). So-called type 2 theta activity (5–9 Hz), also known as "immobility theta", is resistant to urethane and is a dominant hippocampal rhythm during aroused immobility (Sainsbury and Montoya 1984). Spontaneous oscillations in the delta and theta range have been previously recorded in the amygdala of urethane anesthetized and behaving mice, respectively (Seidenbecher et al. 2003; Bazelot et al. 2015) [for review (Bocchio et al. 2017)]. Furthermore, single units in BLA, vHC and PFC of behaving rats fire at frequencies below 4 Hz (Sotres-Bayon et al. 2012). In the NAC of awake freely-moving mice spontaneous activity is in the theta-delta range with a peak at 1–2 Hz (Luo et al. 2018). We noticed a difference in the PFC LFP power spectrum between the BLA–NAC–PFC and vHC–NAC–PFC experimental condition. We can rule out an anesthesia effect, because in both conditions animals were anesthetized in the same way and showed comparable breathing rates (data not shown). Although, we cannot provide a full explanation for this difference, we speculate that it might be due to layer-specific differences in PFC. A layer-specific LFP power has been reported by Bastos et al. (2018) in frontal cortex recordings of monkeys. Their results show that the LFP power in frequencies below 20 Hz is stronger in deep layers than in superficial layers. Given this finding, we speculate that the LFPs in BLA–NAC–PFC versus vHC–NAC–PFC condition may have been recorded in different layers of PFC. We cannot fully exclude that LFPs in BLA–NAC–PFC were mostly recorded in superficial layers whereas LFPs in the vHC–NAC–PFC condition mostly in deep layers of PFC. However, our data on the spontaneous LFP activity in BLA, NAC, vHC and PFC are in good agreement with these previous observations, supporting the assumption that the light urethane anesthesia used in our experiments has only mild effects on the spontaneous network activity.

### Correlation of spontaneous activity between two brain regions

A number of our observations are in good agreement with previous reports confirming strong interactions between specific brain regions within the cortico-limbic network. Furthermore, we provide novel insights into the functional connectivity in different frequencies ranging from delta to beta. Using spike count cross-correlation, we determined the coupling strengths between two connected brain regions. Consistent with previous studies (Pitkänen et al. 2000; Seidenbecher et al. 2003; Hoover and Vertes 2007; Stujenske et al. 2014; Hultman et al. 2016; McGarry and Carter 2017; Burgos-Robles et al. 2017), all five pairs that we tested were closely connected and revealed spike-count correlations. The *r*_*sc*_ in our results compared to previous studies that measured spike count correlations (Cohen and Newsome 2008; Leavitt et al. 2013; Taub et al. 2018) were smaller. However, these previous studies measured the correlation between neurons located in the same brain region, close to each other, and having similar stimulus preferences in response to stimulus presentation or during performing a task (Cohen and Kohn 2011). In our data we observed a high variability in the lag time of cross-correlations between different pairs. Therefore, we decided to obtain the directionality information of connections using tPDC analysis. Although spike cross-correlation has been used extensively for functional connectivity analysis and in some cases for inferring the directionality of connections, it might not be an informative method of analysis to reveal the direction of connection across areas with bidirectional connectivity, which is the case for the brain regions that we examined in our study (Russo and Nestler 2013). As Bastos and Schoffelen (2016) discuss in their review article, the spike cross-correlation analysis could be informative for those regions with a dominant unidirectional connectivity (e.g. the retino-geniculate feedforward pathway; see Usrey et al. 1998), while this might not be the case for regions with a bidirectional connectivity as in our study.

### Functional connectivity in cortico-limbic network

In agreement with previous observations, all five pairs that we examined in the present study showed synchrony in multiple frequencies [for review (Bocchio et al. 2017)]. As shown previously (Dzirasa et al. 2011a, b; Hultman et al. 2016), we identified the delta and theta band as the dominant synchrony rhythms in the BLA–NAC–PFC network. Moreover, our results provide evidence for the contribution of a third brain region on the pair synchrony of two connected areas. Specifically, more than half of the observed coherence in the BLA–NAC is mediated by the common PFC input, indicating a powerful impact of PFC in enhancing the BLA–NAC synchrony. A similar influence of PFC in synchronizing BLA and NAC was observed for the vHC-NAC pair in vHC–NAC–PFC recordings, albeit at a smaller intensity (31% vs. 60%), such that more than one-third of the vHC–NAC synchrony magnitude could be explained by the PFC influence. Our results also revealed the implication of NAC in cortico-limbic synchronization, although its magnitude differs for different paired areas. While NAC accounts for a remarkable amount of the BLA–PFC coherence, its influence is less pronounced in vHC-PFC synchrony such that the majority of vHC–PFC synchrony (~ 70%) is preserved after removing the NAC contribution. Given this finding and previous observations (Siapas et al. 2005; Wierzynski et al. 2009; Godsil et al. 2013; Padilla-Coreano et al. 2016; Liu and Carter, 2018), it is most likely that vHC and PFC synchrony is mediated through a direct connectivity, although the role of an unrecorded area cannot be excluded. In contrast to vHC-PFC, NAC-PFC synchrony is remarkably dependent on the input from a third connected area. Indeed, nearly half of the observed coherence in NAC-PFC could be explained by BLA influence. Altogether, our results indicate that all four regions contribute in vHC-BLA-NAC-PFC network synchrony and PFC slow waves are the key player in this synchronization (Karalis et al. 2016; Hultman et al. 2016).

### Directed connectivity in cortico-limbic network

**Fig. 8:**
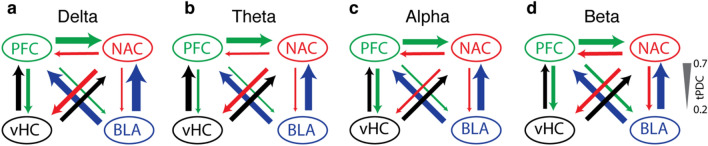
Summary diagram of directed functional connectivity in the cortico-limbic network for spontaneous activity in the delta (**a**), theta (**b**), alpha (**c**) and beta (**d**) frequency band. Thickness of arrows indicates normalized values of tPDC strength (see scale right)

In addition to describing mutual synchronicity, we determined the direction and strength of information flow for each pair in the cortico-limbic network (Fig. 8). For all five pairs, we found reciprocal connections, which is consistent with previous studies (Little and Carter 2013; Senn et al. 2014; Hultman et al. 2016; Reppucci and Petrovich 2016; Klavir et al. 2017; McGarry and Carter 2017). However, the strength of the information flow, depending on target area, was weighted toward either directions. In particular, BLA appeared to be as the principle source of information flow in the BLA-NAC-PFC network, as the connectivity from BLA to NAC and PFC was remarkably stronger than that from NAC and PFC to BLA. These results might be sound in contrast to our results in coherence, where PFC has been suggested as a key structure in BLA-NAC-PFC synchrony. However, it should be considered that our results in mutual synchrony can only describe the existence of synchrony for each pair and determine the contribution of a third recorded area (Muthuraman et al. 2008) and the direction of information flow cannot be resolved by relying merely on coherence analysis. In contrast, tPDC analysis provides the complementary information and particularly the direction of connection (Muthuraman et al. 2018).

Unlike BLA, the NAC implication in this network seems to be more as a “receiver”, as the strength of NAC output to BLA and PFC was much weaker than the inputs from BLA and PFC to NAC. This finding is consistent with previous observations on NAC innervation (Phillipson and Griffiths 1985). Unlike NAC and BLA, PFC behaves differently in its interaction with NAC and BLA, functioning as a “donor” in communicating with NAC and as a “recipient” in communicating with BLA. This finding is in line with our partial coherence results where the 60% of BLA–NAC synchrony is mediated by PFC. Our findings, therefore, might be supporting evidence for the established regulatory role of PFC in regulating limbic dynamics (Hultman et al. 2016; McGarry and Carter 2017), although our study cannot answer this question directly.

Our results in cortico-limbic network show that vHC is highly interconnected with PFC and that this connection is bidirectional. Although this is in contrast with the thoughts of unidirectional connectivity from vHC-PFC (Xu et al. 2019), the interplaying of hippocampus and PFC in memory has been also reported by other studies (Preston and Eichenbaum 2013), supporting bidirectional connectivity in vHC-PFC. Although the strength of information flow from vHC to PFC was stronger than PFC to vHC in theta rhythm, for other frequencies (delta, alpha and beta) there was no significant difference between information flow from vHC to PFC and from PFC to vHC, indicating bidirectional connectivity for vHC and PFC, at least in LFP activity. This is consistent with the previous report on interplaying hippocampus and PFC in memory (Preston and Eichenbaum, 2013).

Unlike vHC-PFC, we did not find any frequency-specific difference directionality for vHC-NAC communication, as the strength of connection from vHC to NAC was comparable to the strength of connection from NAC to vHC. In the delta band, NAC was influenced mainly by PFC rather than vHC, which is in line with the known role of the PFC slow wave in regulating limbic areas (Karalis et al. 2016; Hultman et al. 2016). In conclusion, our results provide insights to the functional connectivity in the cortico-limbic network by determining the strength and direction of information flow within this network and implicating PFC as a key structure.

### Limitations

Our results in cortico-limbic functional connectivity in lightly anesthetized mice have a few limitations. First, although our anesthesia protocol consisting of a combination of a very low dose of urethane and adding a sedative was designed to reduce the adverse effects of urethane on brain state as much as possible, the influence of even this light anesthesia on our results should not be neglected and therefore a direct translation from our results to awake state is difficult. Second, we did not study the BLA-vHC communication, due to technical difficulties for recording these two areas simultaneously using our multi-site MEA approach. However, the interaction of BLA and vHC and its relevant role in emotion, cognition and psychiatric disorders has been probed in previous studies [for review (Bocchio et al. 2017)]. Third, we did not include gamma band activity in our tPDC analysis, as activities in the gamma range were rather small. However, previous studies in mice demonstrated gamma band alterations and implications in anxiety-related disorders (Popescu et al. 2009; Dzirasa et al. 2011b). Using our experimental approach, the role of gamma rhythms in the cortico-limbic network may be elucidated in behaviorally challenged animals. Fourth, we did not investigate the architecture of functional connections, which would have provided complementary insights into specific interaction between areas. Indeed, using a correlation matrix the topology of functional connections can be examined and a graph theory (Rubinov and Sporns 2010) can be employed to describe the connection organizations in the network.

## Conclusion

In conclusion, we have characterized the functional connectivity in the cortico-limbic network of lightly anesthetized wild-type mice. Performing simultaneous recordings in multiple brain regions by using multi-channels probes, we found that BLA, NAC, PFC and vHC are highly interconnected and synchronized in their spontaneous activity. We also determined the implication of each region in synchronizing the other areas of the network. Our results revealed the powerful role of the PFC in synchronizing the cortico-limbic network, which is consistent with previous findings. Given the observed bidirectional connectivity in all five tested pairs, we measured the strength of both feedforward and feedback connections by applying a time-resolved partial directed coherence analyses, as a powerful computational tool, to our data. We found that in the BLA–NAC–PFC network, BLA serves as a principal source of information flow, NAC functions more as a “recipient” and PFC acts as a “donor” for NAC and as a “recipient” for BLA. We also found that in the theta band the strength of information flow from vHC to PFC is stronger than in the opposite direction. Our results obtained in lightly anesthetized wild-type mice provide further information on the physiological connectivity of the cortico-limbic network and represent the basis to study alterations in this network in animal models of psychiatric disorders. We hypothesize that the connectivity between these regions may be altered in mouse models of psychiatric disorders (Hultman et al. 2016; Karalis et al. 2016; Padilla-Coreano et al. 2019).

## Supplementary Information

Below is the link to the electronic supplementary material.

Supplementary file1 (DOCX 704 KB)
