# Supplementary material for: Functional and directed connectivity of the cortico-limbic network in mice in vivo
Source: Brain Struct Funct. 2021 Jan 13;226(3):685–700. doi: 10.1007/s00429-020-02202-7 (PMC7981333; doi:10.1007/s00429-020-02202-7)
Supplement: Supplementary file 1 — Supplementary file1 (DOCX 704 KB) [file 429_2020_2202_MOESM1_ESM.docx]

**Supplementary Figure**

**Supplementary Fig. 1.** Localization of brain regions of interest in adult mouse brain atlas (Paxinos and Franklin, 2001). A represents anterior, P represents posterior. Distances from Bregma in mm.
